# Machine Condition Monitoring System Based on Edge Computing Technology

**DOI:** 10.3390/s25010180

**Published:** 2024-12-31

**Authors:** Igor Halenar, Lenka Halenarova, Pavol Tanuska, Pavel Vazan

**Affiliations:** Institute of Applied Informatics, Automation and Mechatronics, Faculty of Materials Science and Technology in Trnava, Slovak University of Technology in Bratislava, 812 43 Bratislava, Slovakia; lenka.halenarova@stuba.sk (L.H.); pavol.tanuska@stuba.sk (P.T.); pavel.vazan@stuba.sk (P.V.)

**Keywords:** edge computing, sensors, predictive maintenance, monitoring, system

## Abstract

The core of this publication is the design of a system for evaluating the condition of production equipment and machines by monitoring selected parameters of the production process with an additional sensor subsystem. The main positive of the design is the processing of data from the sensor layer using artificial intelligence (AI) and expert systems (ESs) with the use of edge computing (EC). Sensor information is processed directly at the sensor level on the monitored equipment, and the results of the individual subsystems are stored in the form of triggers in a database for use in the predictive maintenance process. The whole solution includes the design of suitable sensors and of the implementation of the sensor layer, the description of data processing algorithms, the design on the communication infrastructure for the whole system, and tests in the form of experimental operation of the device in laboratory conditions. The solution includes the visualisation of the production system status for the operator using an interactive online map.

## 1. Introduction

The last decade has been a challenging period for modern industrial enterprises, with the production process being significantly influenced by two key factors. The first is the implementation of new approaches in automation, which has led to the emergence of concepts such as Industry 4.0 and Industry 5.0 [1].

This method of production entails the active utilisation of contemporary technologies, including machine learning (ML), big data (BD), and digital twin (DT) technologies, in conjunction with novel modern communication methodologies, namely wireless communication and the Internet of Things (IoT). Enterprises that have been implemented in accordance with these concepts are referred to as cyber-physical systems [2] (source 2). The prevailing trend is the introduction of novel approaches in line with Industry 5.0, such as human-centricity, personalisation, and sustainability.

The second factor is the increased pressure on production efficiency, which has the effect of reducing the energy intensity of the production process and lowering the price of the final product. It is evident that these trends are frequently in conflict with one another, and the desired outcome can only be achieved through a substantial transformation of the methodology employed in production. One area where there has been a discernible shift in approach is in the maintenance of existing equipment. In an optimal scenario, this process entails the maximisation of the utilisation of production equipment, with a concomitant reduction in the frequency of breakdowns and periods of downtime due to malfunctions. The establishment of a continuous functional state of production equipment hinges upon the implementation of an effective maintenance mechanism.

Traditional maintenance methods, which include both corrective and preventive maintenance, are insufficient for the effective minimisation of equipment downtime. Consequently, the prevailing trends offer companies a novel direction in the domain of maintenance, utilising a predictive methodology [1]. This is exemplified by predictive maintenance (PDM) [3] and condition-based maintenance (CBM) [4]. The PDM concept employs a variety of techniques to anticipate future failures. The available literature indicates that PDM technology can be broadly classified into two main categories [5]. Firstly, there are model-based methods for predictive maintenance that rely on stochastic processes, including Gaussian processes, Markov processes, Poisson processes, and Weibull distributions [6,7,8]. The second approach, which has gained greater popularity in recent times due to the advent of artificial intelligence, is data-driven predictive maintenance. This approach to PDM employs machine learning or deep learning methods to predict the health state and remaining useful life of a system’s operation, negating the necessity for prior knowledge of the equipment’s condition and its degradation process [5,6,7].

A second approach to predictive maintenance is CBM. This concept is frequently employed in conjunction with prognostics and health management (PHM) [9]. There are a number of international standards that pertain to CBM. The most widely recognised standard is International Organization for Standardization (ISO) 13374 [10]. The most prominent implementation and functional specification of this standard is the Open System Architecture for Condition-Based Maintenance (OSA-CBM), which is held by Machinery Information Management Open System Alliance (MIMOSA) [11].

The resulting platform is a de facto standard that encompasses all functional requirements pertinent to this domain, including data collection and the recommendation of maintenance actions. The OSA-CBM architectural framework comprises seven layers, as outlined by MIMOSA [12], although the seventh layer is the application interface. The model is shown in Table 1.

The objective of this contribution is to develop a condition monitoring system for real-world production systems, which could potentially be integrated into a PDM system. The implementation comprises sensor phase selection, data transformation, and the creation of a data visualisation proposal with alert generation for the operator. In accordance with the presented OSA-CBM architecture, it can be categorised within layers one, two, three, and seven.

The utilisation of edge computing technology enables the implementation of the entire process at the lowest possible level, namely at the stage of data collection from the monitored process. Consequently, the initial stages of the monitoring process, namely data collection, data processing and transformation, and data evaluation, are conducted practically directly at the sensor level. The principal advantage of this solution is the minimal load on the communication infrastructure and the low demands on the database server.

The remaining portion of the publication is organised as follows: The following section presents an overview of the most relevant concepts from the field of process condition monitoring. The Section 3 of the publication presents a detailed account of the design of the system’s overall architecture, including the creation of the sensor layer and a comprehensive description of the architectural modules associated with data collection and analysis. The Section 4 provides a comprehensive description of the implementation environment for the proposed system and a detailed account of the interface implementation for the operator and, potentially, the PDM system, including outputs from experimental operation. Finally, the Section 5 of the publication presents a comprehensive evaluation of the proposal and the achieved results in the conclusion.

## 2. Related Work

To prevent breakdowns in the production process, a variety of maintenance strategies are employed at different levels of complexity. These include breakdown or corrective maintenance (maintenance performed on equipment that has malfunctioned), time-based or preventive maintenance (maintenance performed on equipment based on a calendar schedule), and predictive maintenance strategies. In an optimal scenario, the zero-defects strategy can be achieved [13].

The PDM process is contingent upon the quantity and quality of the information and data obtained from the production process, particularly in the context of data-driven predictive maintenance. One of the fundamental aspects of this technology is the monitoring of equipment and process anomalies, the diagnosis of degradation states and faults, and the prediction of the evolution of degradation to failure. This enables the estimation of the remaining useful life. This paradigm, designated prognostic and health management (PHM) [14], is employed to facilitate both condition-based and predictive maintenance. The process of PHM itself comprises three principal tasks: fault detection (anomaly detection), fault diagnostics (degradation level assessment), and fault prognostics (remaining useful life prediction) [14]. It is evident that the entire process is inherently intricate. Consequently, the objective of this endeavour is to discern anomalies within the production process, specifically within the fault detection phase. This process can be disaggregated in accordance with the OSA-CBM model, comprising data collection, data processing, and data evaluation phases, culminating in fault detection and alert generation.

The preliminary stage is the deployment of sensors, which results in the formation of a sensor layer over the monitored process. Modern systems are typically equipped with a number of sensors. An additive sensor layer for monitoring mechanical systems may comprise the monitoring of fundamental physical quantities, including the temperature of selected parts and components, the current consumption of electrical elements, and pressure.

The measurement of temperature in mechanical components offers two key advantages: firstly, the relative ease of measurement, and secondly, the importance of temperature data in the identification of faults. Accordingly, temperature is identified in numerous publications as a factor conducive to the identification of potential faults.

In the work of Bora et al. [15], a proposal for the detection of faults in reciprocating compressors is outlined, employing the integration of temperature data with machine learning algorithms. The findings of this study indicate that the proposed model is capable of anticipating the status of the device over a 10-day period, utilising solely temperature sensor data and an ARIMA learning module on a Raspberry Pi.

In their work, the authors Yang et al. [16] describe thermal-error-related signals of the spindle system together with motor current, and propose an intelligent monitoring system for the CNC machine. Similarly, the authors Krawczyk and Szuba [17] propose a predictive maintenance system that includes artificial intelligence, which is designed to predict the necessity of maintenance or repairs of switch gears by analysing temperature changes under varying current loads.

Muneeshwari et al. [18] employed temperature sensors on machinery, in conjunction with vibration, pressure, humidity, and accelerometer sensors, to develop an Internet of Things (IoT)-driven predictive maintenance model. Furthermore, temperature sensing is employed in extreme conditions to anticipate the deterioration in performance of a gas turbine, as illustrated in the work of Dai et al. [19]. They put forth a data-driven methodology to forecast equipment condition utilising an autoregressive neural network. This trend is similarly observed in other domains, such as agriculture [20].

The other physical value, which is relatively straightforward to discern, is pressure in technical media distributions. This is applicable in a multitude of settings, predominantly those involving the pressure of air (though not exclusively), where the pressure energy of compressed air is transformed into mechanical work in automation.

The issue of pressure sensing in gas is discussed in the publication by Yun et al. [21]. The authors conducted an analysis and comparison of various simulation-based prediction models for predicting the pressure in gas regulators, with notable outputs that are generally applicable for gas pressure monitoring. A collective of authors, Kumar et al. [22], address a similar topic. The authors examine the potential of artificial neural networks when integrated with the finite element method and employed as predictive tools for anticipating the failure pressure of pipelines. From the perspective of automation and the focus of the article, the objective is to prevent the failure of air distribution systems, which are composed of valves, pipes, and cylinders. This problem area is addressed in the core publication of authors Yuan et al. [23]. They propose a fault classification model based on convolutional neural networks (CNNs) for various types of air cylinders, which are widely used in industrial robotic hands in automation systems, from assembly lines to mechanical manufacturing lines.

Liang et al. [24] present a compressed air station pressure monitoring system based on the LoRa protocol. They propose a data monitoring platform that can monitor and evaluate the pressure information of hyperbaric equipment in real time through a wireless sensor network with alarm triggers.

More complex are the procedures of vibration analysis, oil analysis (including oil temperature, viscosity, and level), and, for rotational components, rotational parts parameters [25,26,27]. In the case of vibration analysis, the complications are a consequence of the more challenging measurement process. Mafla-Yépez et al. [28] present a number of strategies for the predictive maintenance of compression ignition engines based on the analysis of vibration data. In his work, he addresses the issue of the pre-established acquisition parameters, which include the sampling frequency, time interval between captures, and the number of samples. The same problem is described by Coelho et al. [29] in a different context. The article proposes a PDM system for industrial equipment using machine learning algorithms with a dataset based on the initial features, such as temperature, operating speed, vibration, and pressure.

A comparable methodology for the monitoring of production equipment parameters is a well-established approach that has been employed in a number of other works and industrial contexts, including chemical industry [30,31], aviation systems [32,33], additive manufacturing [34,35], the energy industry [36], and even agriculture [37,38].

The subsequent phase, in alignment with the MIMO model (layers 2 and 3), entails a data manipulation process accompanied by preprocessing for analysis and data evaluation with alert generation. This stage of the solution represents the objective of this proposal in principle. The preparation of data for analysis is a relatively straightforward process, dependent on the type of sensor employed (analogue or digital) and the methodology employed for the interpretation of the measured value.

The process of identifying anomalies from data patterns is considerably more complex. Anomalies are defined as patterns in data that deviate from the expected norm. These patterns can manifest in a variety of forms, including point anomalies, contextual anomalies, and collective anomalies. It is often the case that anomalies represent the sole indication of performance deficiencies discernible within data sets [29]. Furthermore, the data collected can vary considerably in type and format, and may be highly correlated, large-scale, and characterised by a high level of noise. The generation of alerts, or error triggers, can therefore be a challenging process, requiring the use of advanced machine learning techniques, such as deep learning networks, convolutional networks, and other data manipulation and data identification processes, including those related to “big data” and “data mining” [39].

Mohammadi et al. [40] presented a comprehensive overview of the various deep learning architectures and algorithms employed in data analytics within the domain of sensing devices. The publication offers a valuable summary of the most commonly used DL models for sensor data processing, a review of practical DL approaches and use cases, and an identification of the challenges and future research directions in the field of DL for sensing systems. The work is limited by its status as an overview, lacking a concrete practical example.

Yang et al. [16] describe an intelligent sensing system for CNC spindles based on multi-source information, including spindle temperature, spindle thermal deformation, operating parameters, and motor current. In order to identify errors in the spindle (and thus generate error triggers), a radial basis function network is employed.

Krawczyk et al. [17] employ a range of machine learning algorithms, including nearest neighbours, linear SVM, RBF SVM, Gaussian process, decision tree, random forest, neural net, AdaBoost, naive Bayes, QDA, and linear discriminant analysis, to demonstrate the potential for power rails failure prediction based on temperature changes. However, a limitation of the study is that some of the presented algorithms were trained exclusively on synthetic data. A comparable approach to data evaluation and fault identification utilising AI is outlined in several other publications [18,22,23,25,27,31,41].

However, there are also solutions that do not employ such intricate mathematical models when generating fault triggers from the collected data. In such instances, it is more efficacious to utilise a condition tree or an expert system (ES) [42]. The deployment of the ES in addressing the issue of anomaly identification and subsequent generation of alerts based on sensor data offers the following advantages: cost reduction, lower equipment load, capacity to operate with incomplete and uncertain information, and simplicity of use.

In a recent study, Liang and colleagues [24] investigated the efficacy of a wireless sensor system based on LoRa communication for predicting failures and abnormal functions in high-pressure equipment. They proposed a real-time pressure monitoring and early warning wireless sensor system based on LoRa communication, with the objective of predicting failures and abnormal functions in high-pressure equipment. The proposal entails an analysis of data from the sensor layer, comprising the pressure value, battery power, temperature, and number of data transmissions at each monitoring point. This analysis is conducted in accordance with pre-established thresholds and rules, which then trigger the alarm mechanism.

In their study, authors H. Lu et al. [43] present an online fault diagnosis system based on an expert system. The proposed fault diagnosis system, which incorporates automated data processing and analysis, is capable of quickly identifying and locating faults, thereby reducing the time required for maintenance. Rojek et al. [44] describe an adaptive 3D printing system with an artificial intelligence model that can be customised using an expert database script. The hybrid model has the potential to markedly enhance the efficiency of the 3D printing process, reducing the necessity for trial and error, minimising material waste, and optimising production time.

Wang et al. [45] put forth a methodology for the identification of underlying correlations amongst multiple sensors and the detection of data patterns from all correlated sensor data over time. This methodology is founded upon the integration of the autoregressive integrated moving average method and an expert knowledge system.

In their study, Zhang et al. [46] present a fault diagnosis approach based on ESs for use in industrial process control systems, as applied to pneumatic actuators. This contribution introduces an algorithmic approach based on a modified expert system that incorporates particle swarm optimisation. It is evident that ESs have a wide range of applications in the process of data evaluation in system monitoring and fault detection, as outlined in a comprehensive analysis of typical ES applications [47].

The previously described methods of data processing are distinguished by a high level of computational intensity. The current trend is towards processing unstructured data at lower levels. Edge computing (EC) [48,49,50] appears to be an appropriate means of data processing at the initial stage, at the lower level. In conjunction with unsupervised neural network technologies, such as convolutional networks [51,52,53], or analogous techniques [54], EC represents a promising avenue for data processing, even in the context of condition monitoring [55]. When monitoring physical variables in a control process, it is frequently the case that a continuous analogue signal must first undergo a digitisation process with high-quality sampling. It is evident that this process generates a considerable amount of data pertaining to the monitored process, yet the information value derived from it is limited. The storage of such voluminous data in a central repository for subsequent processing gives rise to an increased demand for storage systems, and traditional data management systems are unable to accommodate the storage and analysis of such data. In order to facilitate central processing, it is necessary to utilise big data technology [39]. The principal advantage of processing such vast quantities of data (structured or unstructured) via EC is the considerable reduction in storage necessities and the capacity to make determinations within the central management system based on pertinent information regarding the monitored process. A disadvantage is the need to utilise smart devices with sufficient computing power at the point of data collection. Nevertheless, the total amount of computing power required for data processing remains constant, but is distributed across multiple layers, resulting in a reduction in the load on the communication infrastructure and storage.

## 3. Materials and Methods

The design of the solution described in this paper is physically implemented in a laboratory environment on a model of a real production line. The line model is created from real components used in commercial industry. One advantage of this solution is that the testing phases of the proposed algorithms and procedures are not limited by production, as in a real environment. This method of implementation allows sufficient time for technology development. Furthermore, in the case of deployment of the developed system in practice, adaptation is quick and easy.

### 3.1. Technical Equipment Used

**Figure 1:**
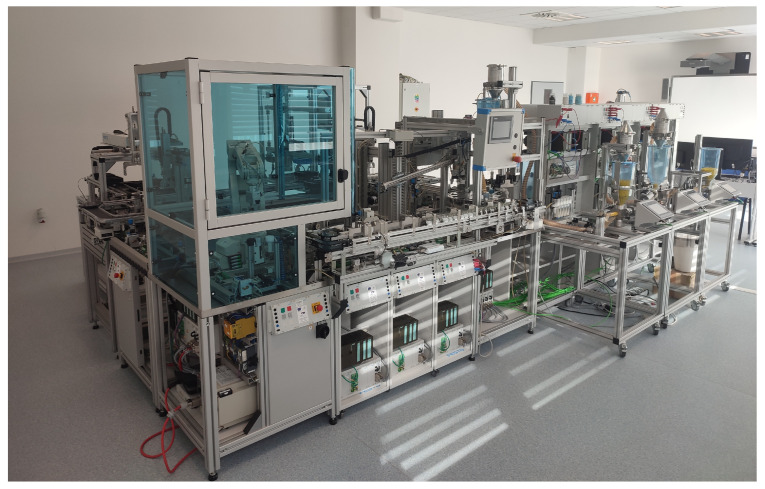
The production line in laboratory environment.

The apparatus employed for research purposes is located within the faculty laboratories. The picture (Figure 1) provides a visual representation of the modular hybrid automatic production system.

The system is composed of the following components:

- Production;
- Manipulation with seals;
- Packing line;
- Filling line;
- System of conveyors;
- Automatic shelf stacker/storage;
- Inputs/outputs.

Moreover, the system incorporates both procedural and factory automation. The device is capable of functioning as a unified complex entity, yet it can also be disassembled into discrete systems. The control system of the separate modules currently comprises an industrial PLC mounted on a bar, connected to regulated technology via 24 pin 488 bus connector, and to a visualisation through an external interface. Operator panels (HMI) are mounted on a plate with outsourced connectors for communication interfaces.

The communication system for the transfer of information within the production line is implemented using the Transmission Control Protocol/Internet Protocol (TCP/IP) and the Ethernet protocol, with a transfer speed of 1 Gbit. In order to enhance data security, the entire line environment is isolated from the external environment by means of the Scalance S612 industrial firewall. Access from the external environment is permitted via a virtual private network (VPN) connection. To enhance security, devices are addressed by static addressing within the private address range 192.168.10.0/24, with a network address translator system implemented on the firewall. For the communication of wireless elements and devices, the Scalance W788 device (Manufactured by Siemens, Munchen, Germany) is utilised in access point mode, with security provided by WPA2 encryption.

**Figure 2:**
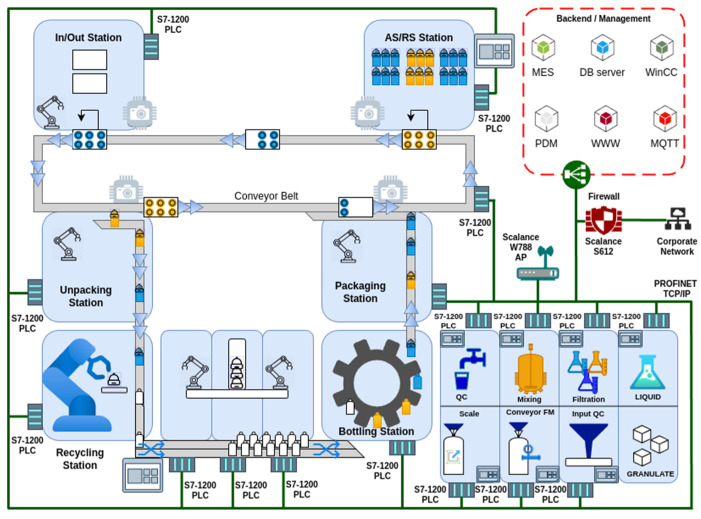
Schematic representation of the production line.

The final product of the production line is a pallet of bottles, the contents of which are mixed in a variable manner according to the recipe, combining granulate and liquid. The entire system (Figure 2) is managed by an MES (Manufacturing Execution System) based on the AVEVA Manufacturing Execution System [56]. The process is controlled by a programmable logic controller (PLC). The aforementioned MES is also equipped with the functionality to monitor the quality of both processes and products, including the automated execution of quality sample plans and the utilisation of statistical control methods. Nevertheless, the aforementioned elements are not yet fully operational.

The proposed system represents an initial step towards the implementation of PDM. In the initial phase, the objective is to establish the infrastructure for the sensor layer and implement the fundamental components for the intelligent assessment of data from the production line environment using EC.

**Figure 3:**
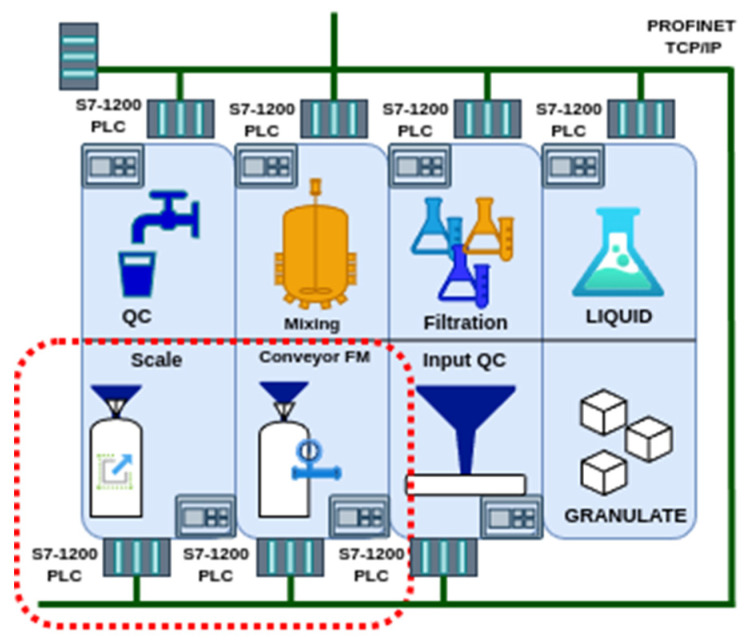
Specification of the location of the equipment used.

For the purposes of this publication, the two modules of the production line have been selected for testing the suitability of the proposed hardware for the anticipated use. These are the workstations located at the inlet of the granulate into the system, specifically the “Conveyor FM” and the “Scale” (Figure 3).

Their selection is based on their suitability for the initial validation of procedures and ideas pertaining to the implementation of the sensor layer. This is due to their simplicity and the limited number of active elements.

The device used to evaluate the data from the sensor layer is the Revolution Pi (RevPi). It is an open, modular industrial PLC based on the Raspberry Pi, and is powered by an ARM Cortex-A7 processor (Arm Holdings plc Cambridge, England). The base unit can be extended with other I/O modules (analogue or digital) and numerous communication interfaces, including Profinet, Profibus, and RS-485. The platform, which is based on Linux, allows for the utilisation of technologies such as Node-RED, Python, and the C language, as well as the execution of applications that are supported by MQTT and OPC UA communication [57].

**Figure 4:**
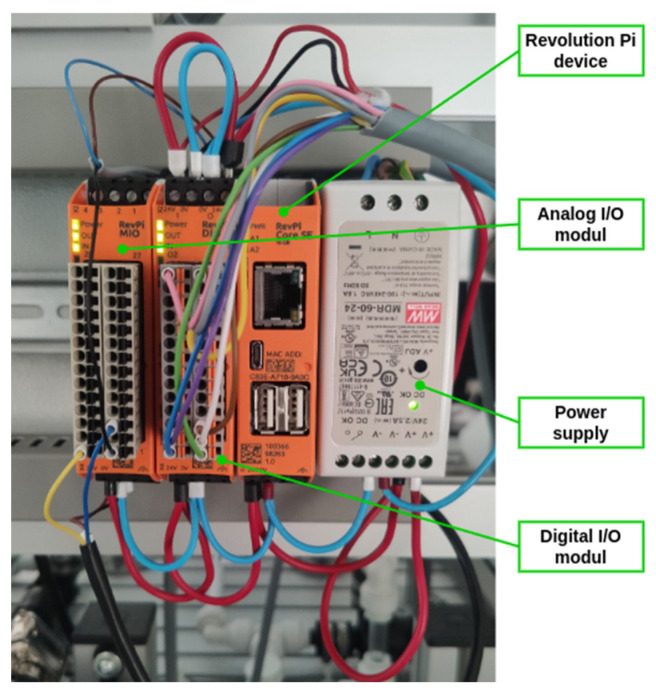
Detail of the device used to control the collection of data.

For the intended use, it was necessary to expand the base unit with an I/O module (RevPi MIO) to connect the sensors (Figure 4). The mentioned device provides sufficient connectivity for the proposed sensors and computing power for processing information from the sensors of both workstations (Figure 3) within the EC.

### 3.2. Sensors Proposal

The design of sensors is based on a synthesis of literature sources, personal experience, and the physical nature of the devices being monitored. This approach allows for the capture of additional information about the state of the device, which is of particular importance when the sensed values are of a complex nature.

#### 3.2.1. Sensor Set for the Conveyor FM Monitoring

**Figure 5:**
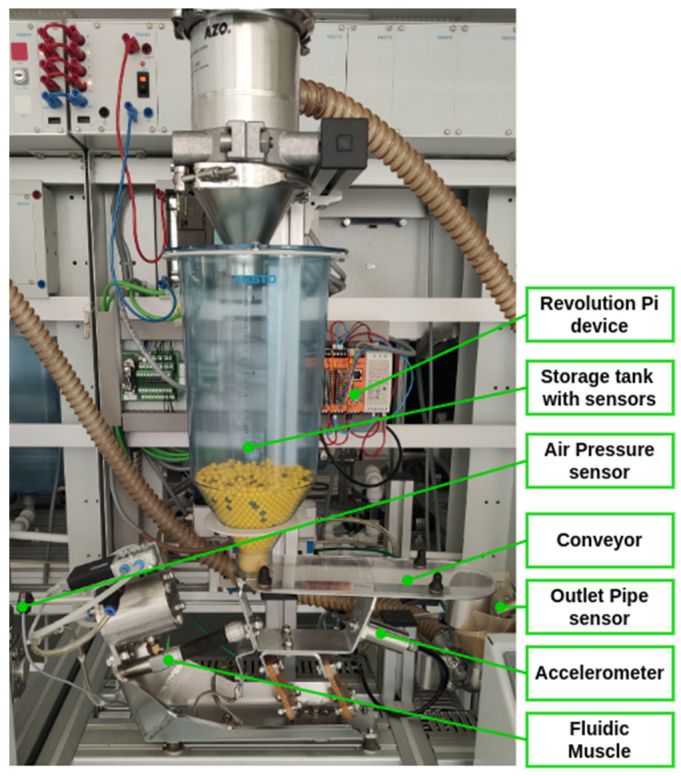
Description of the Conveyor FM workplace.

The device may be described as a vibration conveyor, which is responsible for the transportation of bulk materials between stations. The apparatus comprises a material storage tank and a fluidic muscle (FM), which serves as the primary source of power for the transfer of materials to the subsequent station. The conveyor is equipped with three sensors for monitoring the material status. Two of these are located in the inlet tank, while the third is located on the outlet pipe to detect when it is full. The outlet pipe can be emptied using air pressure, which is controlled by the outlet in automatic or manual mode. The active components (air control valves) are controlled by PLC. Therefore, the entire device is simple yet functional. It should be noted that there is no information available regarding the status of the key elements for PM, including the condition of the driving element (FM), air pressure (intake valve status), and the condition of the conveyor itself (movement possibility). The detailed description is provided in Figure 5.

In order to gain additive data from the production process, three additive sensors were used in accordance with the controlled process and the simplicity of the testing purposes. An air pressure sensor was employed for the purpose of monitoring the status of the air within the system, while an accelerometer (vibration sensor) was utilised for monitoring the conveyor’s state. Additionally, temperature monitoring was conducted. The aforementioned temperature sensor comprises two sensors, one measuring room temperature and the other measuring the internal temperature of the device. This sensor is employed solely for the purpose of monitoring the CPU load in the RevPi device, which is used for data processing. The data obtained from the temperature sensor can be readily processed, whereas the other two require more complex techniques. In our proposal, the air pressure sensor is designed to measure not only the actual value of pressure in the system, but also the course of pressure in the system.

The SPTE-P10R-S4-V-2.5K air pressure sensor, produced by the FESTO company (Festo SE & Co. KG, Esslingen, Germany), will be utilised for this purpose (see Figure 5). The selected sensor [58] has an appropriate pressure range (0–1 MPa) and an appropriate output range (0–10 V), and is compatible with the existing line equipment.

To measure vibrations, an accelerometer is employed, specifically the low-cost industrial grade IP67 sensor RS-WZ3/WZ1-*-1 (Shandong Renke Control Technology Co., Ltd., Jinan China), which is based on MEMS technology. The main technical parameters, including vibration velocity accuracy (within ±1.5% of the full-scale reading (@1 kHz, 10 mm/s)) and frequency range (10 Hz to 1600 Hz), are appropriate for the intended use. Figure 5 illustrates the placement of the sensor on the conveyor device.

#### 3.2.2. Sensor Set for the Scale Monitoring

**Figure 6:**
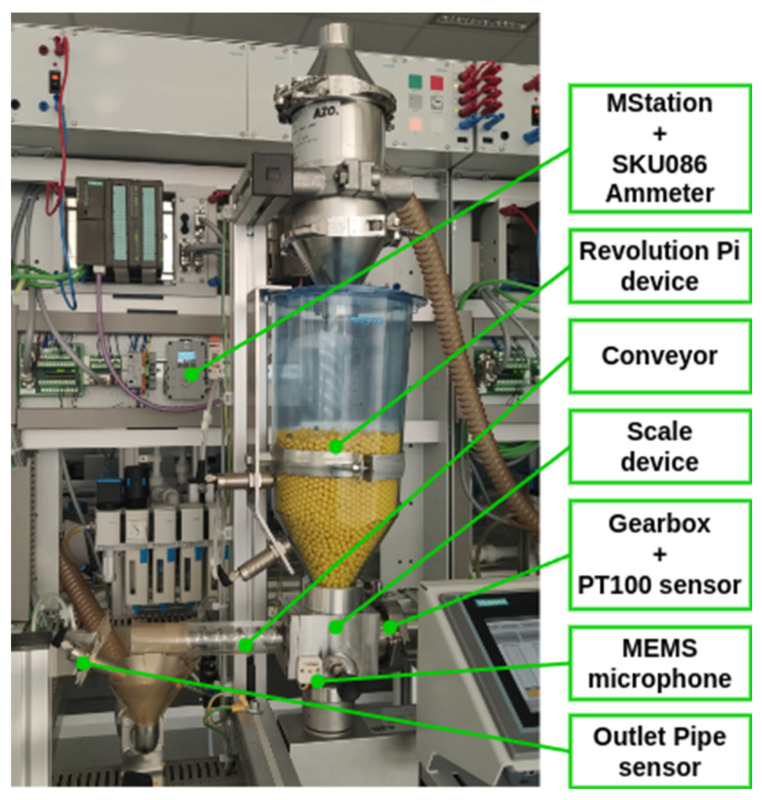
Description of the Scale workplace.

The device (Figure 6) has been designed with a similar configuration to that of the previous workstation. The objective of the device is to quantify the volume of bulk material delivered to the process. The device comprises two principal components: a material storage tank and a scale with a screw conveyor, powered by a DC motor (BG65X25SI, produced by Dunkermotoren Gmbh, Bonndorf, Germany). As with the preceding model, the device is equipped with three sensors for monitoring the material level: two situated in the input reservoir and one for detecting the filling of the outlet pipe. The outlet pipe can also be emptied using air pressure, which is controlled by the outlet in automatic or manual mode. The active components, namely the air control valves, are controlled by a PLC.

The supplementary data for monitoring the system status can be classified into two categories in accordance with the particulars of the process. The driving force behind the transfer of the granulate at the inlet and outlet of the workstation is the pressure of the air. The primary component of the PDM is the conveyor drive, which comprises a DC motor and a gear mechanism. It is proposed that the following additional sensors be employed. In the case of sensing the condition of the electric motor, gearbox and bearings, based on literature sources [59], it is proposed that the following parameters be monitored:

- Air pressure;
- Vibrations;
- Gearbox temperature;
- Motor current load.

For the air pressure monitoring, the same sensor as in the previous case, the SPTE-P10R-S4-V-2.5K, is used.

The implementation of vibration sensing in the gear mechanism differs from that observed in the Conveyor FM workplace. A digital MEMS silicon-based microphone [59] that utilises pulse-density modulation (PDM) signalling is employed as the sensor. This microphone is renowned for its high signal-to-noise ratio, high sensitivity (94 dB SPL @ 1 kHz, −22 dBFS), low power consumption (600 µA), resilience to RF interference, and smooth frequency response [60].

The temperature sensor for the conveyor drive, which is used to monitor the temperature of the gearbox, can be readily addressed through a cost-effective and straightforward approach. The PT100 sensor [61], which has a temperature range of 0 °C to 150 °C and a measurement precision of ±0.2% F.S., is an ideal solution. Its operational range and accuracy are sufficient for the intended application.

The final parameter to be monitored is the current load. The utilised motor, BG65X25SI [62], is a brushless direct-current (DC) motor with an integrated encoder, capable of transmitting 4096 signals for speed control. From the perspective of electrical consumption monitoring, the following parameters are of particular significance: rated voltage 24 V, maximum current 4 A. To measure the current, the SKU086 [63] metre unit is employed. The rationale for selecting this sensor is based on its suitability for the specified current range, I2C bus communication, integrated I2C isolator (CA-IS3020S), and accuracy (0.1% FS, ±1 count) and resolution (0.3 mA).

**Figure 7:**
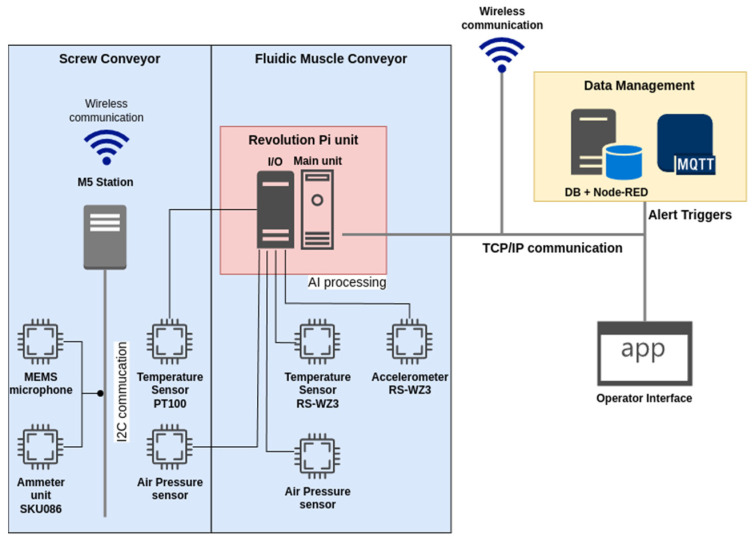
Sensor connection scheme.

The air pressure sensor and the temperature sensor are directly connected to the RevPi device on the analogue input. The remaining sensors (MEMS microphone and current sensor) (M5Stack Technology Co., Ltd, Bao’an District, Shenzhen, China) are connected due to the technical sensing process using the M5Station operator station (M5Stack Technology Co., Ltd, Bao’an District, Shenzhen, China) [64], which is a multipurpose industrial-level programmable embedded controller with Espressif ESP32 SOC, integrated Wi-Fi solution, dual-core low-power Xtensa^®^ 32-bit LX6 microprocessor. The station runs a service program to capture data in set cycles and preprocess it for fault identification. Communication between the M5Station and RevPi is wireless. The wiring diagram of the sensors is shown in Figure 7.

The air pressure sensor and the temperature sensor are directly connected to the RevPi device on the analogue input. The remaining sensors, namely the MEMS microphone and current sensor, are connected using the M5Station device from M5Stack [64], which is a multipurpose industrial-level programmable embedded controller.

The M5Station is equipped with an Espressif ESP32 system-on-chip (SoC), an integrated Wi-Fi solution, and a dual-core low-power Xtensa^®^ 32-bit LX6 microprocessor (M5Stack Technology Co., Ltd, Bao’an District, Shenzhen, China). The station runs a service program to capture data at set intervals and preprocess it for the identification of faults. Communication between the M5Station and RevPi is wireless.

### 3.3. Neural Network Description

Neural networks are widely used as classifiers for image recognition due to their inherent characteristics. There are numerous types of neural networks, and in this case, a multilayer perceptron neural network is employed. This is a feedforward artificial neural network with a backpropagation algorithm for training the network. Such neural networks are commonly utilised to address problems that necessitate supervised learning, as well as research in computational neuroscience and parallel distributed processing and man.

To facilitate the implementation process, the Matlab R2024 software was employed for the design and testing of the neural network (NN).

The algorithm employed is an iterative learning algorithm for artificial neural networks, developed by Moller [65]. It is based on conjugate directions and is distinguished by the absence of a linear search at each iteration to determine the step size or learning rate, in contrast to other conjugate gradient algorithms that necessitate a linear search at each iteration. This makes the algorithm more expeditious than others [66]. In the Matlab environment, function trainscg permits the training of any network, provided that its weight, net input, and transfer functions possess derivative functions. Backpropagation is employed to calculate the derivatives of performance with respect to the weight and bias variables [67].

As indicated in the literature [66,67], the step size in this algorithm is a quadratic approximation function of the error function, thereby enhancing its robustness and independence from user-defined parameters. The step size is estimated using a distinct criterion, wherein second-order information is employed to accelerate the convergence rate by repeatedly calculating the gradient at each iteration. The second-order term is calculated as follows:

(1) $${\bar{S}}_{k}=\frac{\dot{E}({\bar{w}}_{k}+\sigma_{k}{\bar{p}}_{k})-\dot{E}({\bar{w}}_{k})}{\sigma_{k}}+\lambda_{k}{\bar{p}}_{k}$$

The $\lambda_{k}$ is a scalar quantity that is adjusted at each iteration in accordance with the sign $\sigma_{k}$:

(2) $$\alpha_{k}=\frac{\mu_{k}}{\delta_{k}}=\frac{{-\bar{p}}_{j\;}^{T}{\dot{E}}_{qw\;}({\bar{y}}_{1})}{{\bar{p}}_{j\;}^{T}\;\ddot{E}(\bar{w}){\bar{p}}_{j}}$$

where:

- $\bar{w}$ is the weight vector in space *Rn*;
- *E*$\left(\bar{w}\right)$ is the global error function;
- $\dot{E}(\bar{w})$ is the error gradient;
- ${\dot{E}}_{qw}({\bar{y}}_{1})$ is the quadratic approximation of the error function;
- ${\bar{p}}_{1},{\bar{p}}_{2},\ldots {\bar{p}}_{n}$ are the set of non-zero weight vectors [67].

The advantage of this approach is that the computer platform utilised as the hardware is directly supported within the Matlab environment. Furthermore, the programs created and exported from Matlab for Raspberry Pi are fully compatible with the used platform and can be directly run.

## 4. System Proposal

The fundamental tenets of data evaluation are as follows. The data are evaluated on the device in a suitable manner and the resulting processing output is stored on the server. In terms of OSA-CBM architecture, the proposed system encompasses the initial three layers, wherein all three layers—the DA layer (access to physical sensor data), the DM layer (data manipulation and preprocessing for analysis, signal/data transformations), and the SD layer (data evaluation, set thresholds/operational limits)—are physically realised in the form of EC and implemented using software within the Revolution Pi unit. The remaining OSA-CBM layers (HA, PA and AG) are not currently addressed, and operator interaction is conducted manually. This issue will be addressed in future research. The stored data are accessed by the visualisation subsystem in the initial phase for the purpose of visualising the status in a manner that is intelligible to the operator. Subsequently, in later phases, the data are accessed for the purpose of meeting the PDM needs. The specific form of processing employed is contingent upon the nature of the data being processed. It is acknowledged that the data will be processed in such a way that the output from the sensor EC layer for each parameter will be presented as information in the form of an alert, which will act as a trigger in the logic state. The logical “0” (FALSE) is used to indicate that no problem has been identified. Value logical “1” (TRUE) indicates the potential existence of an issue on a specific device.

The processing method is contingent upon the specific type of parameter being monitored. Considering the need to minimise the CPU power required to obtain fault state information from the measured values, the following assumption is made. The initial group contains the measured values. The directly measured quantity is sufficient for the acquisition of fault information. Following transformation, the data are interpreted as an alert trigger directly, preferably without processing. The second group consists of those parameters where the fault can be identified by the EC. In this case, the fault information is processed using a set of rules. Finally, those parameters where the fault information can be identified from the time course of the measured values are processed using a suitable kind of neural network, with an output of logical “0” (FALSE) in case of a no fault detected or logical “1” (TRUE), meaning an identified fault.

**Figure 8:**
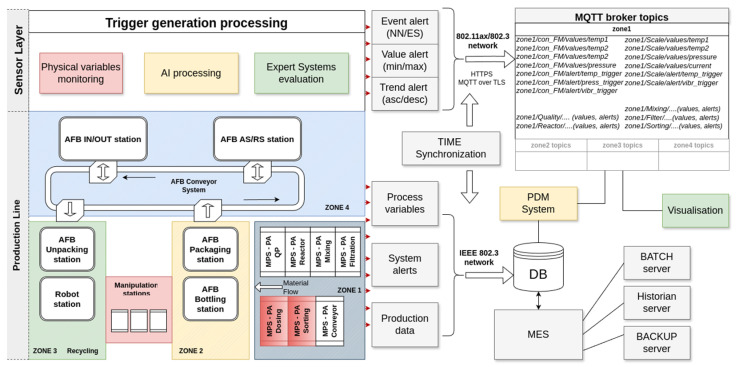
Schematic representation of the proposed system with MQTT subsystem.

All data are processed at the lowest layer in the form of EC using RevPi or, eventually, other smart sensors. The outputs of the individual processes are directly stored on the MQQT broker, which is located within the control server pool. The individual processes store the outputs as a publisher to a prescribed queue. The output visualisation application (subscriber) accesses the data on the MQTT, while the visualisation is implemented using the NODE-RED framework. A schematic representation of the whole system is shown in Figure 8.

The data communication within the system is facilitated by the TCP/IP protocol via metallic cabling in the case of RevpPi, whereas in the case of intelligent sensors, it is achieved through a wireless medium.

One advantage of this solution is that it allows for the reduction of the load on the communication infrastructure with respect to sensor data. The default cycle time of the utilised device (RevPi) is less than 10 milliseconds, with a typical value of 8 milliseconds. The analogue-to-digital converter (ADC) utilised for data acquisition from the analogue input is of the 16-bit wide [57]. When continuous sensing of parameters (especially waveforms of values in the form of time-dependent curves) is employed, the volume of data at a sufficiently high sampling rate is considerable, particularly when one considers the fact that for the entire system dozens of sensors are utilised within the line.

The proposed architectural solution aims to minimise the data flow by processing the data at the sensor level. The processing results are stored exclusively in the form of triggers within the MQTT.

**Figure 9:**
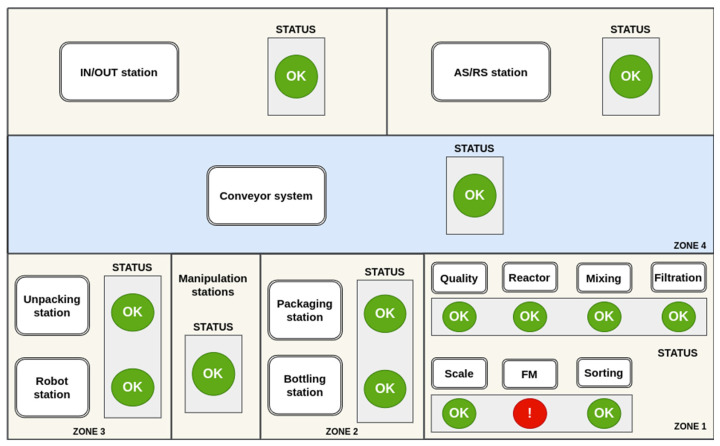
Main screen of proposed application.

A visualisation system implemented in the Node-RED environment [68] is used to display the detected errors. The application (Figure 9) is designed with a user-friendly interface for the operator. The basic screen presents a graphical representation of a simplified diagram of the entire production line. The input data for the application are alerts in the form of triggers stored within each topic on the MQTT server. These alerts are indicated by an alert icon within the basic screen interface.

**Figure 10:**
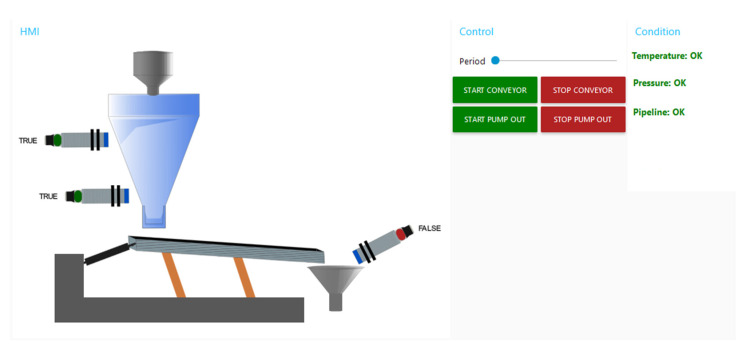
Conveyor FM workstation management screen.

The principal benefit of employing Node-RED is the straightforwardness with which it can be displayed across disparate devices, solely via a web browser. This obviates the necessity for installing an application on the device. Moreover, in the case of touch screens, the operator is able to directly control the communication interface through touch. Once a device with an identified alert has been selected (by clicking on the alert icon), the operator is then able to view detailed information for each device and analyse the alert in more detail based on which measurement it was generated from. Figure 10 illustrates the control interface of the Conveyor FM workplace. The operator can then activate direct control of the device, check the current process data, or open the alert history record. The process control is realised directly by communication on the specific PLC.

## 5. Practical Experiments

Although the project is implemented in a simplified environment in laboratory conditions, the implementation of the entire system for monitoring the condition of the production line is a highly complex matter with many different procedures. In the initial phase, only two workstations are implemented out of the total of 16 within the production line: Conveyor FM and Scale. This section provides examples of generating alerts based on the values measured by sensors at these parts.

In general, however, implementation practices can be classified into three principal categories. The initial category is the measurement of the temperature of selected elements, wherein the actual measured value or gradient is of primary importance. In this instance, the generation of triggers is conducted in a simplified manner during the initial phase of the project, based solely on the measured temperature of the equipment and the defined gradient. This is carried out with due consideration of the overall thermal conditions within the laboratory environment. Similarly, the evaluation of the motor load current measurement is addressed in the case of a screw conveyor.

The generation of alerts in the context of device temperature monitoring is achieved through the implementation of a condition cycle, which is both straightforward and effective. The temperature of the key elements and the actual ambient temperature are monitored, and the measured values are stored in a database within the RevPi device. An error trigger is generated in two distinct scenarios. In the initial scenario, an error is triggered when the maximum permissible discrepancy between the specified temperatures is surpassed. In the second case, an error is generated when the maximum defined temperature is exceeded, irrespective of the ambient temperature. In the present implementation, errors are displayed exclusively as a warning icon within the main application environment. The operator can ascertain the current values via the menu of the specific device. Similarly, the evaluation of the motor current load is conducted, whereby a condition cycle is employed to ascertain the current value and generate an alert in the event of a maximum value being exceeded. A more complex approach has been taken to identify alerts in the case of air pressure monitoring in the system, whereby accelerometer data and vibration recorded by a MEMS microphone are processed.

In addition to the transportation of the granulate, the primary source of energy for movement in the case of fluidic muscle at the Conveyor FM workstation is air. In this case, the actual value of the measured pressure is not the sole indicator used to identify a fault (leak) in the compressed air piping; rather, a simple EC is implemented in the form of knowledge represented by a condition tree.

**Figure 11:**
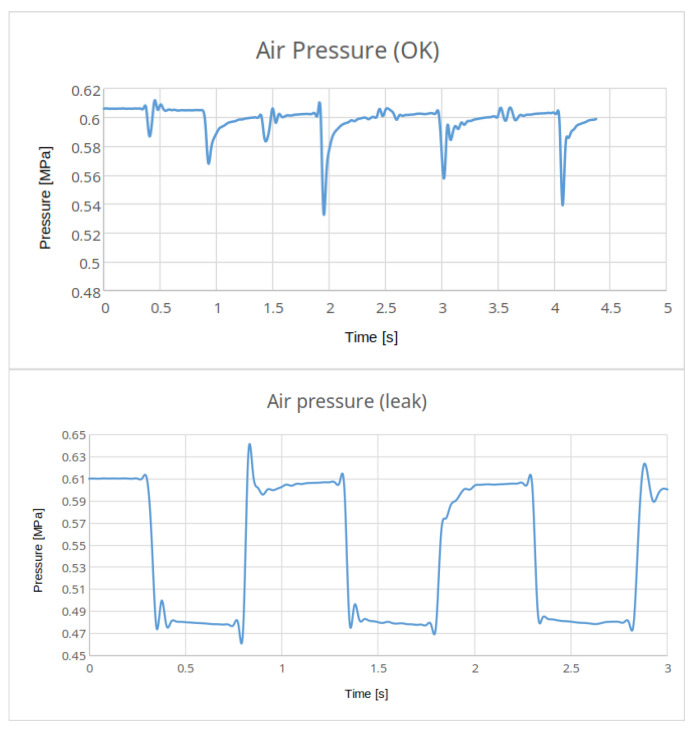
Air pressure graph examples.

Figure 11 illustrates the air pressure waveforms during normal operation in the event of an air line fault associated with a reduced air supply volume in the distribution system. It is evident that with partial duct damage, the actual conveyor function process remains operational, and no errors are detected by the PLC. However, the graph clearly indicates a change in the operating parameters.

As the fluidic muscle operates in regular cycles, the error information is an output from the algorithm for processing pressure over time, rather than a mere measurement taken from the pressure sensor. The employed algorithm must be straightforward to reduce the computational complexity of assessing a potential error in the intake manifold. The following procedure is employed for the purpose of detecting the aforementioned fault.

The fundamental premise is that the air pressure within the pneumatic system, following a duty cycle in normal conditions, will stabilise at the same value as in the previous cycle. In the event of leakage, however, there will be a discrepancy in the values at which the pressure stabilises. The program-controlled measurement is conducted upon the activation of the vibratory conveyor. Initially, the pressure value at which it stabilises during the initial phase of the conveyor cycle is recorded, followed by the pressure value at which it stabilises during the subsequent phase.

Upon activation of the switchboard, the current air pressure is promptly measured and duly recorded in the database. Upon deactivation of the switchboard, a subsequent measurement of the air pressure is conducted, and the recorded values are then subjected to a comparative analysis.

The evaluation process is as follows:

- If the measured air pressures differ from each other by more than 0.01 Mpa, then a fault attribute is stored in the database;
- The values of five consecutive work cycles of the air pressure are compared;
- If an evaluation of five consecutive cycle checks results in an indication of a failure condition, the error trigger on the pressure pipe is set to TRUE and the trigger is stored to MQTT topic;
- The application will display an “Air leak” error icon on the user interface.

The measured values are stored in the database in the RevPi device for only a brief period. Despite its simplicity, this system is capable of effectively detecting potential leaks in the distribution system.

**Figure 12:**
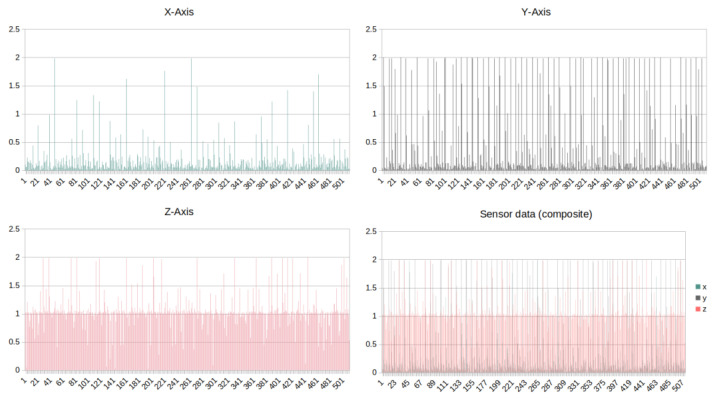
Accelerometer data in three dimensions X/Y/Z.

To identify the appropriate function of the fluidic muscle, the movement of the conveyor is monitored using an accelerometer. The device utilised (Figure 5) measures values in three axes—X/Y/Z, as illustrated in Figure 12.

Given the manner in which the sensor is affixed to the conveyor body, the most significant measurement is that taken along the Y-axis. The data are collected and stored in the database in a synchronous manner with the conveyor duty cycles, undergoing subsequent processing.

The objective is to utilise a neural network to ascertain whether the process is continuously operational and the values are within the specified tolerance limits, with an output of “TRUE” or “FALSE” (0/1). The process evaluation is conducted at regular intervals, and the data are separated into patterns that are suitable for processing using the proposed neural network.

**Figure 13:**
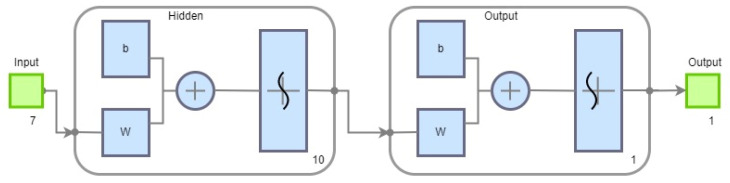
The used NN architecture.

To minimise the computational demands on the EC, the neural network must be simple and effective. Accordingly, a three-layer neural network was constructed, as shown in Figure 13. The input layer comprises seven neurons, which correspond to patterns derived from the recorded data stream. The network contains one hidden layer of 10 neurons. The entire network is implemented as a two-class classifier, with an output layer comprising a single neuron. The identification of a successful FM stroke in the data pattern is represented by the logic value of the output neuron, which assumes the values “0” or “1”.

**Figure 14:**
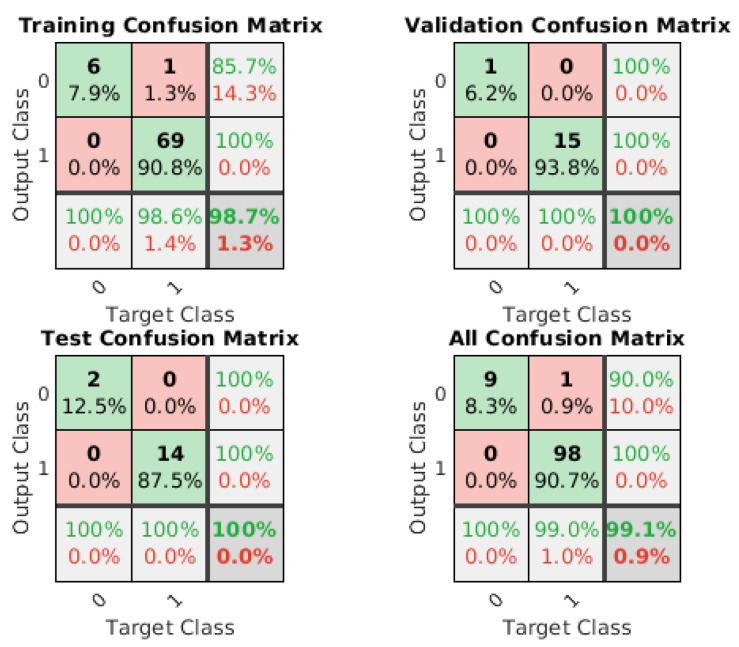
Confusion matrix of the trained NN.

Following a series of trials conducted within the Matlab environment, it was determined that the scaled conjugate gradient algorithm (TRAINSCG function) yielded the most optimal outcomes in this particular instance (Figure 14).

**Figure 15:**
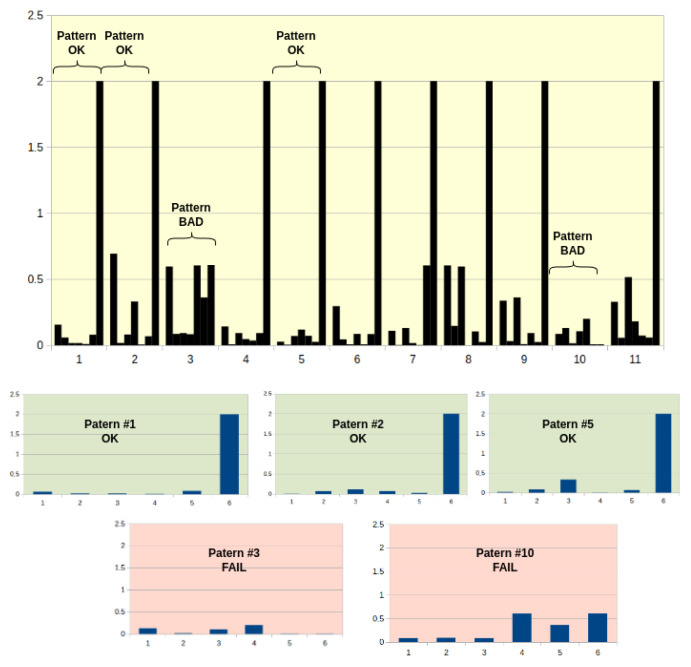
Data patterns for evaluation by NN.

The technical solution within the EC is as follows: the application for communication with the sensor and data preparation for NN evaluation (splitting to patterns—Figure 15) is running in RevPi and has been written using the Python language.

The process of the neural network is isolated, and the resulting output is stored in a MQTT topic and presented in the application as an alert icon, eventually with a green or red colour message in a more detailed view.

The data gathering process at the other workplace, which employs a screw conveyor and scale, is analogous to the aforementioned process. It is designed to monitor four parameters: air pressure, temperature, current consumption, and gearbox vibrations.

In this instance, the crucial factor in anticipating potential malfunctions is the discernment of the vibrations captured by the MEMS microphone situated on the gear body. Prior to identifying a potential fault and triggering an alert, it is essential to implement a process whereby the microphone recording is subjected to spectral analysis using a Fourier transform, with the objective of determining the dominant frequencies of the signal. The program for the physical implementation of the measurement and frequency decomposition is implemented in Python and runs within the M5 Station at regular time intervals. It records samples with a duration of one second, a sampling rate of 16 kHz, and a data width of 16 bits. Subsequently, the frequency domains are identified, and the recorded audio is then compared to the stored inspection pattern.

**Figure 16:**
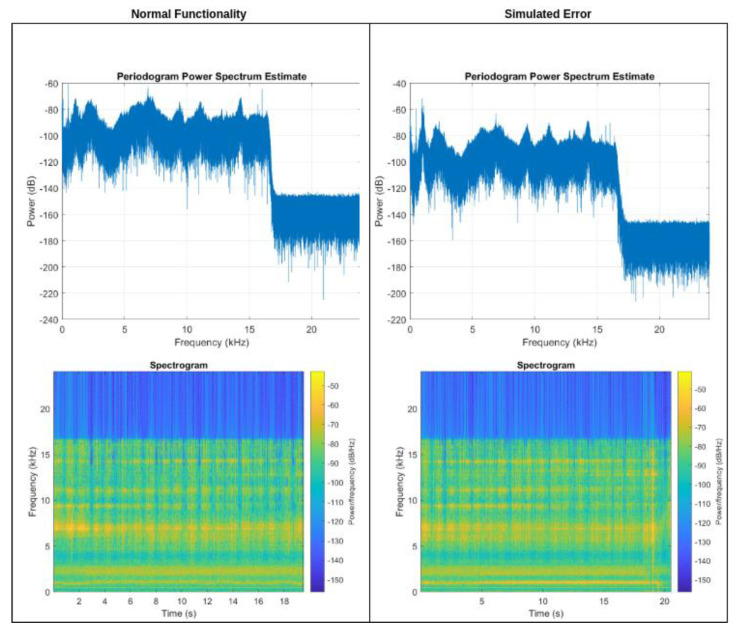
Spectral analysis of MEMS microphone data from gearbox.

The pattern is shown in Figure 16, located on the left side (Normal Functionality). In the event of the detection of a non-standard frequency within the recorded audio, an alert trigger is generated. From the perspective of the project, it should be noted that the FFT analysis shown in Figure 16 is relatively simple and cannot cover all cases that may occur in real operation. However, this feature is not yet fully implemented and will be addressed in the next phase of the project.

The remaining data on the current consumption and gearbox temperature are merely supplementary in nature and serve only to facilitate the identification of a potential fault. The generation of alerts for these parameters is achieved through the recording of the current value at regular intervals, with subsequent comparison to historical values. As the frequency analysis of the recorded sound increases, so too does the importance of collecting these parameters and identifying anomalous values. It is assumed that the bearing failure identified by isolating the non-standard frequency in the sound recording is related to the increased resistance in the rotation of the mechanism, which in turn causes a rise in temperature and energy consumption in the mechanism.

## 6. Conclusions

The subject matter addressed in this article is particularly relevant in the current era, given the growing implementation of Industry 4.0 and 5.0 in technical practice. The detection of equipment and production system conditions represents a highly complex domain, largely due to the multitude of technologies employed for the collection and processing of data from production systems. The objective of this thesis is to develop a system for machine condition monitoring based on edge computing technology. This study has identified the fundamental physical variables that are essential for the identification of machine condition. Furthermore, the requisite hardware components for the deployment of the sensor layer, along with the communication protocol between the constituent elements, have been proposed. The primary components outlined in the publication encompass the formulation of a data transformation methodology and the delineation of multiple avenues for acquiring device state information. These are classified according to the type of physical quantity, the method of representation, and their relevance to the monitored process. In the final section, examples of critical state identification using ES and NN technologies are provided, including practical examples and outputs of the implemented experiments. It should be noted that this is the initial phase of the project. In subsequent stages, it will be necessary to implement a similar condition monitoring system for all components of the production line and to extend the current sensor and diagnostic system to include the capability of monitoring all parts of this line. The actual implementation process of the resulting PDM system will be the subject of further research in the future. However, it can be stated that the experimental solution described in the paper is able to identify an emerging fault on a device within the monitored system. The disadvantage is that it is designed directly for a specific device.

The proposed solution’s primary contribution is the integration of contemporary trends, such as machine learning and edge computing, with practical applications. The deployment of EC for data processing facilitates the direct processing of data at the lowest process level, while the supervisory system interacts with the final outputs of the EC and neural network subsystems. This approach, even with real-time data processing, significantly reduces the burden on the communication infrastructure, lowering the database performance requirements and the load on the PDM system. The proposed sensor scheme, the data method evaluation in the form of trigger generation, and the MQTT system are applicable as an initial stage towards the implementation of a PDM system. Furthermore, the creation of an application to visualise the information obtained would be beneficial for the operator.

The objective of future research is to extend the existing sensor layer and fault identification system to other equipment within the production line. As these workplaces differ from one another, the components of the sensor layer must be designed with particular attention to the specifics of each workplace. In the project’s final phase, the objective is the design of a suitable coupling between the implemented system and a PM system that can accommodate the outputs from the whole implemented system.

The proposed hardware components and data collection methods for process diagnostics, as well as the principles of fault identification, are applicable not only within the model but also to other components of the production line on which the PDM system is implemented. Following adaptation, they can be implemented in other systems in real conditions. It is acknowledged that such a solution would require a significant investment in financial and human resources in a real deployment. Furthermore, the developed alert generation algorithms have the potential to be utilised in other industrial sectors for process health monitoring in general.

## Figures and Tables

**Table 1:** OSA-CBM architecture [12].

Layer	Functional Block	Description
1	Data Acquisition (DA)	Access to physical sensor data.
2	Data Manipulation (DM)	Data manipulation and preprocessing for analysis (signal transformations, data transformation).
3	State Detection (SD)	Data comparison, alert generation (data evaluation, set thresholds/operational limits).
4	Health Assessment (HA)	System’s health computation.
5	Prognostics Assessment (PA)	Prediction of current health into the future (processing of statistical data, historical and collected data).
6	Advisory Generation (AG)	The action suggestion (alternatives, maintenance actions).
7	Human Interface	Data presentation.

## Data Availability

Data are contained within the article.
